# Limitations of Reconstructing Pentacam Rabbit Corneal Tomography by Zernike Polynomials

**DOI:** 10.3390/bioengineering10010039

**Published:** 2022-12-28

**Authors:** Mohamed Baraya, Jessica Moore, Bernardo T. Lopes, Richard Wu, FangJun Bao, XiaoBo Zheng, Alejandra Consejo, Ahmed Abass

**Affiliations:** 1 Department of Production Engineering and Mechanical Design, Faculty of Engineering, Port Said University, Port Said 42526, Egypt; 2 Department of Civil Engineering and Industrial Design, School of Engineering, University of Liverpool, Liverpool L69 3GH, UK; 3 Department of Ophthalmology, Federal University of Sao Paulo, Sao Paulo 04017-030, Brazil; 4 Brighten Optix Corporation, Shilin District, Taipei 11167, Taiwan; 5 Eye Hospital, Wenzhou Medical University, Wenzhou 325035, China; 6 Department Applied Physics, University of Zaragoza, 50009 Zaragoza, Spain; 7 Department of Mechanical, Materials and Aerospace Engineering, School of Engineering, University of Liverpool, Liverpool L69 3GH, UK

**Keywords:** corneal tomography, Pentacam, Zernike polynomials, rabbit eye, curve fitting

## Abstract

The study aims to investigate the likelihood of Zernike polynomial being used for reconstructing rabbit corneal surfaces as scanned by the Pentacam segment tomographer, and hence evaluate the accuracy of corneal power maps calculated from such Zernike fitted surfaces. The study utilised a data set of both eyes of 21 rabbits using a reverse engineering approach for deductive reasoning. Pentacam raw elevation data were fitted to Zernike polynomials of orders 2 to 20. The surface fitting process to Zernike polynomials was carried out using randomly selected 80% of the corneal surface data points, and the root means squared fitting error (RMS) was determined for the other 20% of the surface data following the Pareto principle. The process was carried out for both the anterior and posterior surfaces of the corneal surfaces that were measured via Pentacam scans. Raw elevation data and the fitted corneal surfaces were then used to determine corneal axial and tangential curvature maps. For reconstructed surfaces calculated using the Zernike fitted surfaces, the mean and standard deviation of the error incurred by the fitting were calculated. For power maps computed using the raw elevation data, different levels of discrete cosine transform (DCT) smoothing were employed to infer the smoothing level utilised by the Pentacam device. The RMS error was not significantly improved for Zernike polynomial orders above 12 and 10 when fitting the anterior and posterior surfaces of the cornea, respectively. This was noted by the statistically non-significant increase in accuracy when the order was increased beyond these values. The corneal curvature calculations suggest that a smoothing process is employed in the corneal curvature maps outputted by the Pentacam device; however, the exact smoothing method is unknown. Additionally, the results suggest that fitting corneal surfaces to high-order Zernike polynomials will incur a clinical error in the calculation of axial and tangential corneal curvature of at least 0.16 ± 01 D and 0.36 ± 0.02 D, respectively. Rabbit corneal anterior and posterior surfaces scanned via the Pentacam were optimally fitted to orders 12 and 10 Zernike polynomials. This is essential to get stable values of high-order aberrations that are not affected by Zernike polynomial fittings, such as comas for Intracorneal Ring Segments (ICRS) adjustments or spherical aberration for pre-cataract operations. Smoothing was necessary to replicate the corneal curvature maps outputted by the Pentacam tomographer, and fitting corneal surfaces to Zernike polynomials introduces errors in the calculation of both the axial and tangential corneal curvatures.

## 1. Introduction

The topography of both the anterior and posterior corneal surfaces can be indirectly measured by capturing cross-sectional images using a corneal tomographer. The process includes edge detection and surface reconstruction in order to generate three-dimensional (3D) surfaces from two-dimensional (2D) images. Since what is being measured is not exactly what is being offered by the tomography machine end-user software, digital signal processing (DSP) methods are usually used to reconstruct the eye surface from a finite number of 2D images. The process could also include surface fitting, smoothing and many other approximations. In this context, the Pentacam rotating camera system employs a Scheimpflug system to provide non-invasive images of the anterior and posterior surface raw elevation as cross-sectional views [1]. There are 25 images with a 14.4° gap in standard settings and 50 images with a 7.2° gap in high-resolution (HR) settings. Once images are acquired, these gaps are bridged. Hence, surfaces are processed into corneal feature maps that describe the anterior surface, posterior surface, corneal thickness (pachymetry) and axial/tangential (sagittal) curvatures that vary across the cornea [2]. It is not apparent to the end-user how surface data gaps are bridged and as maps are calculated from the bridged posterior and anterior surfaces, it is key that both researchers and users utilising this device understand the processes that may have been employed. Then, researchers will be able to consider the effect of impeded DSP processes when using tomography-based corneal surface measurements in treatment plans. Additionally, corneal tomography measurements are vital in the diagnosis of keratoconus, monitoring of ectasia progression, and pre and post-surgical assessments [3]. It is, therefore, important that DSP approximations and possible associated induced errors are fully understood.

**Figure 1:**
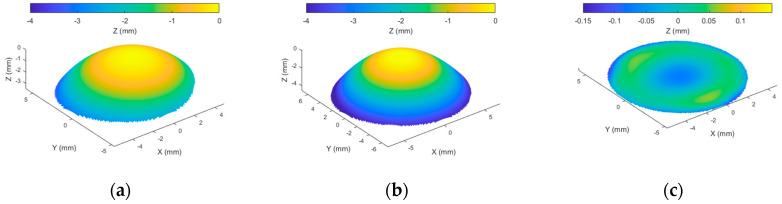
Example of reconstructing anterior corneal surface (**a**) by using third order Zernike polynomial to get the fitted surface in (**b**); however, a surface residual remains without fit in (**c**). Arithmetically, the height of the anterior surface (**a**) equals the height of the fitted surface (**b**) plus the height of the residual surface (**c**).

**Figure 2:**
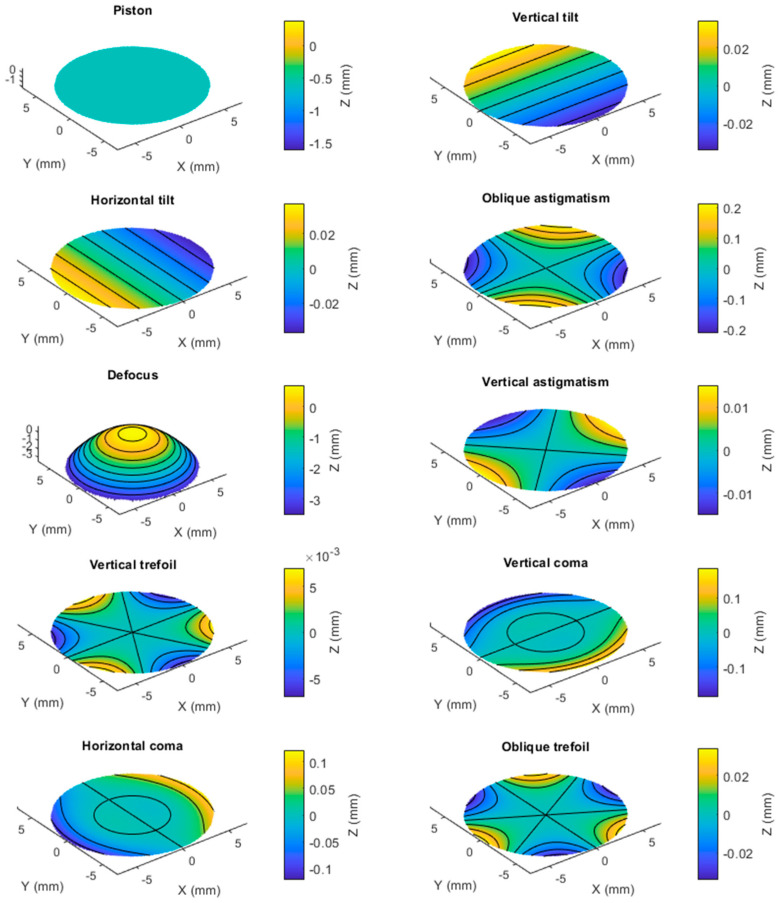
The process of fitting the anterior corneal surface, shown in Figure 1, using 3rd order Zernike polynomial with ten terms. When these terms are added, they reconstruct the surface in Figure 1b.

When a corneal surface is fitted to a Zernike polynomial of any order, it is expected to achieve a fit with residuals unless the surface was already fitted to one of the same orders. As an example, when a right rabbit corneal surface was fitted to a 3rd order Zernike polynomial as in Figure 1, not all surface components were fitted, and considerable residuals remained. This is because adding up the Zernike polynomial terms for this fit (Figure 2) does not fully represent the original surface perfectly. 

In our previous work, Wei et al. [4] conducted an assessment of the capability of Zernike polynomials to correctly reconstruct human corneal surfaces measured by different anterior eye tomography measurement devices, including Pentacam. Their results suggested that Zernike polynomials of orders 12 and 10 provided optimal fitting to the anterior and posterior surfaces, respectively, for healthy and keratoconic human corneas. 

In this animal-based study, in addition to investigating the optimal Zernike polynomial fitting orders on rabbit eyes, further analysis was conducted to improve understanding of any assumptions that are used in the calculation of the corneal curvature maps outputted by the Pentacam corneal tomographer. The potential loss in accuracy that is incurred when Zernike polynomial fitted surfaces are used in the calculation of corneal curvature maps was then investigated. This work further improves understanding of the inner digital signal processing workings of the Pentacam device, and the limitations of Zernike fitted surfaces, which will directly enhance both the clinician and the researcher’s ability to use the data appropriately.

## 2. Materials and Methods

### 2.1. Animal Subjects

**Figure 3:**
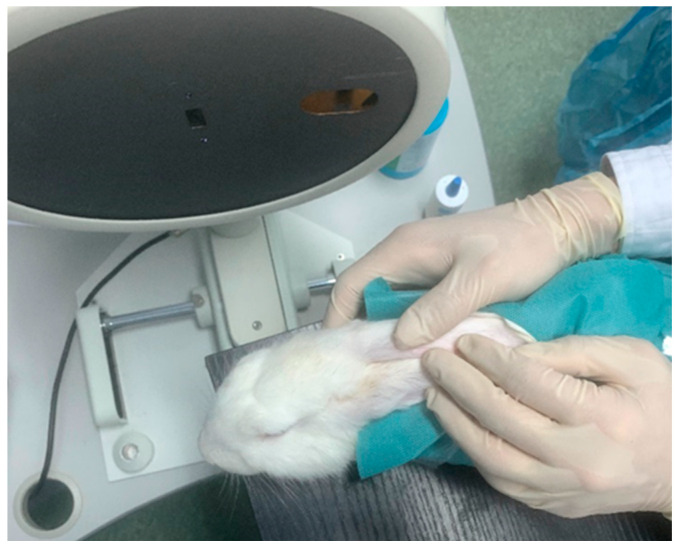
Positioning of a rabbit eye during the Pentacam eye scanning process at Wenzhou Medical University.

Twenty-one Japanese white rabbits (2–3 kg) from the Animal Breeding Unit at Wenzhou Medical University were used in this study in the presence of a veterinarian. All rabbits were treated in agreement with the Association for Research in Vision and Ophthalmology (ARVO) Statement for the use of Animals in Ophthalmic and Vision Research and with the approval of the Laboratory Animal Ethics Committee of Wenzhou Medical University (code: wydw2021-0065). The rabbits had their IOP assessed (mean ± SD = 12.4 ± 1.7 mmHg) after capturing the Pentacam corneal images, using a Tono-pen tonometer (Reichert, Inc., New York, NY, USA) to ensure the eyes were not subjected to elevated IOP. Pentacam measurements were performed in a dim-light room using an adjustable height table and manual positioning to control the rabbit eye location during the eye scanning process, Figure 3.

### 2.2. Data Collection

Clinical tomography data has been collected from both eyes of rabbits using Pentacam (OCULUS Optikgeräte GmbH, Wetzlar, Germany). Raw elevation data collected by the Pentacam for the anterior and posterior surfaces were analysed using a custom-built MATLAB code (MathWorks, Natick, MA, USA). Data were extracted in a cloud of 3D points at locations on a squared mesh grid in both nasal-temporal and superior-inferior directions. The grid considers locations from −7 to 7 mm in both of the principal directions. Raw elevation values that were not part of the cornea were disregarded in this study. 

### 2.3. Corneal Surface Fitting

The quality of fitting Zernike polynomials to a corneal surface was quantified by the root mean squared (RMS) error; the less error, the more accuracy. The term “error” in this context signified the difference in the raw elevation between the clinically measured corneal surface elevation and the Zernike polynomial fitted surface. Consider a surface grid centred around the corneal apex, then the radius of each point on this grid, $\rho_{g}$, is calculated as

(1) $$\rho_{g}=\sqrt{X_{g}{}^{2}+Y_{g}{}^{2}},$$

where $X_{g}$ and $Y_{g}$ represent the coordinates of each of the grid points. 

A normalised form ρ of the radius $\rho_{g}$ is required for Zernike fit, and can be calculated as

(2) $$\rho =\frac{\rho_{g}}{\rho_{max}},$$

where $\rho_{max}$ is the maximum radius observed in the data, which in this case was set to 5 mm to ensure that the data were in the Pentacam’s most reliable measurement area, as peripheral measurements are less reliable. Any surface data beyond this maximum radius were disregarded in these analyses. The Zernike raw elevation $Z_{n}^{m}\left(\rho ,\varphi \right)$ is given by [5]

(3) $$Z_{n}^{m}\left(\rho ,\varphi \right)=\left\{\begin{array}{cc} R_{n}^{m}\cos\left(m\varphi \right) & m>0\\ R_{n}^{m}\sin\left(m\varphi \right) & m<0 \end{array}\right.,$$

where φ is the azimuthal angle of the coordinates $X_{g}$ and $Y_{g}$, n is the radial order of the polynomial, m an azimuthal integer index that varies from −n to n for even (m-n) and equals 0 for odd (n-m) and $R_{n}^{m}$ is a radial polynomial, defined as

(4) $$R_{n}^{m}\left(\rho \right)=\overset{\frac{n-m}{2}}{\underset{k=0}{\sum }}\frac{{\left(-1\right)}^{k}\left(n-i\right)!\rho^{n-2k}}{k!\left(\left(n+m\right)/2-k\right)!\left(\left(n-m\right)/2\right)!}\;\;\;\;\;\;\;\;\left(0\leq \rho \leq 1\right),$$

Zernike raw elevation (height) term $Z_{n}^{m}\left(\rho ,\varphi \right)$ was fitted to the anterior and posterior corneal surfaces exported by the Pentacam software. The RMS error was calculated for every fit as,

(5) $$\mathrm{RMS}=\sqrt{\frac{\sum_{i=1}^{q}{\left(Z_{i\;fit}-Z_{i\;surf}\right)}^{2}}{N}},$$

where $Z_{fit}$ is the Zernike fitted surface height and $Z_{surf}$ is the measured raw elevation surface height and N is the total number of data points considered in the RMS calculation. Pentacam surface data grid is 141 by 141 spaced by 0.1 mm with around 8840 valid measured data points (depending on the quality of measurement) out of the total of 19881 grid points (44.5%). During the fitting process, 80% of the data points were randomly selected for polynomial fitting, and the other 20% were used for the RMS error calculation following the Pareto principle [6]. Using a different set of data points in validation is essential as validating on the original set used in the fitting process overfits this set and leads to misleading small RMS values. Right and left eyes were always treated separately to avoid any possible bias in the results [7,8], and no superior-inferior mirror-imaging data merging techniques were applied in the current study.

It was previously identified that the optimal Zernike order for the anterior surface was 2 orders higher than for the posterior surface [4]. This was considered in this analysis by maintaining a two-order difference between the Zernike polynomials of the anterior surface when compared to the posterior. For example, when fitting the anterior surfaces to an order 5 Zernike polynomial, the posterior surface was fitted to one of order 3. Whilst maintaining this rule, the order of the anterior surface was increased from 3 to 20 and for each order, both the axial and tangential refractive power maps were calculated and compared to the power of the original unfitted maps.

In order to evaluate the effect of fitting order selection in clinical practice, three high-order aberration terms’ coefficients were selected for further investigation. Vertical and horizontal commas are both being used in Intracorneal Ring Segments (ICRS) selection for Keratoconus patients [9], and spherical aberration is being used for pre-cataract operations [10].

### 2.4. Corneal Refractive Power Estimates

The corneal refractive power P was calculated using the Gaussian optics formula [11,12]:

(6) $$P=\frac{n_{cornea}-n_{air}}{R_{anterior}}+\frac{n_{aqueous}-n_{cornea}}{R_{posterior}}-\frac{T_{c}}{n_{cornea}}\times \frac{n_{cornea}-n_{air}}{R_{anterior}}\times \frac{n_{aqueous\;}-n_{cornea}}{R_{posterior}}$$

where the refractive indices of the air, $n_{air}$, cornea, $n_{cornea}$, and aqueous humour, $n_{aqueous}$, were set to 1.0, 1.376 and 1.336, respectively, [13,14]; $R_{\mathrm{anterior}}$ and $R_{\mathrm{posterior}}$ represent the instantaneous radii of curvatures of the anterior and posterior surfaces, respectively; and $T_{c}$ is the central corneal thickness. When analysing the raw Pentacam data, the central corneal thickness, $T_{c},$ the value measured by the Pentacam Scheimpflug system was employed. When Zernike fitted corneal surfaces were considered, $T_{c}$ was calculated by subtracting the Z-axis value of the fitted corneal posterior surface from the fitted anterior surface at the corneal apex. To find the overall refractive power, both the axial and tangential versions of the radii of curvature were considered. Axial curvature $K_{a}=\frac{1}{R_{a}}$ and tangential curvature $K_{t}=\frac{1}{R_{t}}$ were determined using a custom-built MATLAB (MathWorks, Natick, MAJ, SA) program following Klein’s methods [15] as in Equations (7) and (8), respectively;

(7) $$K_{a}=\frac{1}{R_{a}}=\frac{1}{\rho_{g}}\int_{0}^{\rho }K_{t}d\rho_{g}=\frac{dZ_{g}/d\rho_{g}}{\rho_{g}{\left(1+{\left(dZ_{g}/d\rho_{g}\right)}^{2}\right)}^{\frac{1}{2}}},$$

(8) $$K_{t}=\frac{1}{R_{t}}=K_{a}+\rho_{g}\frac{dK_{a}}{d\rho_{g}}=\frac{d^{2}Z_{g}/d\rho_{g}{}^{2}}{{\left(1+{\left(dZ_{g}/d\rho_{g}\right)}^{2}\right)}^{\frac{3}{2}}}.$$

Corneal $R_{anterior}$ and $R_{posterior}$ in Equation (6) were substituted by either axial radius of curvature $R_{a}$ or tangential radius of curvature $R_{t}$ depending on the type of the calculated refractive power map. Z-coordinates were substituted by those of the anterior or posterior surface, depending on the corneal surface where the curvature was being determined. Refractive power errors due to surface Zernike polynomial fittings were calculated for the central optic zone of the cornea up to 3 mm diameter, the average pupil size among normal adults in daylight [16,17].

### 2.5. Smoothing

The axial and tangential power maps were smoothed using the robust discretised smoothing spline method [18]. Different degrees of smoothing were applied using a positive scaling parameter S, with higher S providing a smoother map. The method, which is based on the discrete cosine transform (DCT), works with equally spaced data in two dimensions. As the degree of smoothing is influenced by the smoothing parameter S, it is appropriate to adjust the value of S to achieve the best smooth estimate of the original data whilst also avoiding over-smoothing, where some data features disappear, or under-smoothing, where the digital noise affects the quality of the data. In the current study, S was fixed to 5 with axial maps and 15 with tangential maps, based on the preliminary investigations carried out in [19,20].

## 3. Statistical Analysis

Statistics and Machine Learning Toolbox of MATLAB (MathWorks, Natick, MA, USA) was used to perform the statistical analysis. The null hypothesis probability (*p*-value) at a 95% confidence level was calculated to compare each set of RMS errors obtained when a corneal surface was fitted to Zernike polynomials of successive orders. Initially, the one-sample Kolmogorov–Smirnov test was used to make sure that each set of RMS errors followed a normal distribution, and then the two-sample *t*-test was used to investigate the significance between pairs of data to check whether they were significantly different. Using the Pentacam squared grid of 141 points, nominally 19881 points were tested for each fitting order. As *t*-tests require independence of the measures, and fellow eyes were not analysed together in the current study, the two-sample *t*-test was deemed suitable to determine whether there is a significant difference between the means of two data groups [21]. The test was used several times in this study to evaluate the differences in RMSs for different fitting orders when corneal surfaces and their refractive power were investigated.

## 4. Results

**Figure 4:**
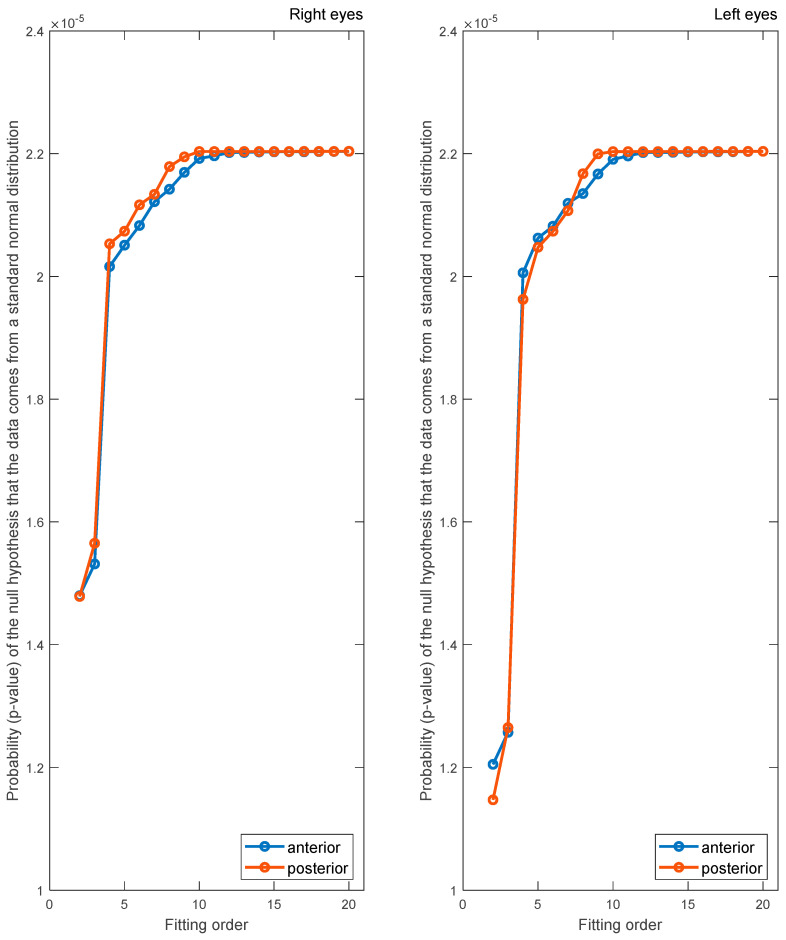
The probability (*p*-value) of the null hypothesis indicates whether the data comes from a standard normal distribution as a result of the Kolmogorov–Smirnov test.

**Figure 5:**
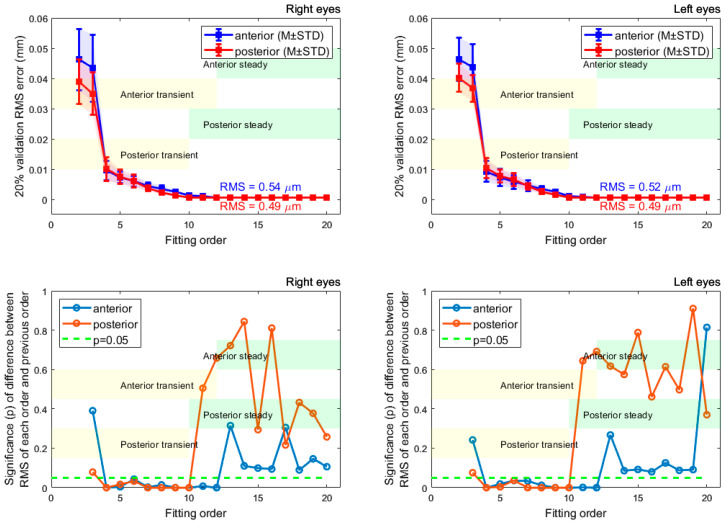
RMS errors for surfaces fitted to different orders of Zernike polynomials are displayed in the first row. Results are shown separately for the right and left eyes. Statistical significance between successive fitting orders RMS values is demonstrated in the second row where the two samples *t*-test were used. The transient state orders show significant changes in power differences; however, steady state orders show a stable change in power differences.

Zernike polynomials of different orders were fitted to the anterior and posterior surfaces of the rabbit corneas, and the corresponding RMS was computed. The Kolmogorov–Smirnov test, Figure 4, confirmed that p-values were under 0.05, indicating that the resulting fitting RMSs form normal distributions. From Figure 5, the anterior and posterior surfaces of the rabbit cornea are best fitted to order 12 and 10 Zernike polynomials, respectively. This is demonstrated in the RMS, which converges to a value close to 0 µm for orders greater than these. The significance was computed for the RMS of successive polynomial orders. This further highlighted the suggested Zernike polynomial orders, as for orders higher than those aforementioned, the difference between consecutive order RMS values became insignificant at a confidence level of 5% (*p* > 0.05). Following convergence of the RMS error, there were residual errors of 0.54 and 0.49 µm for the anterior and posterior surfaces, respectively, in the right eye population and 0.52 and 0.49 µm, respectively, in the left eye population.

**Figure 6:**
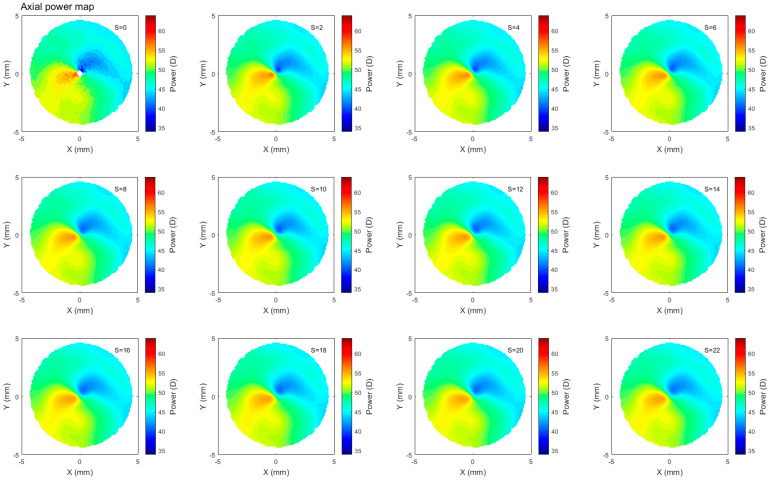
Axial refractive power map of a rabbit’s left eye smoothed to different ranges with the scaler S changing from S=0, which represents no smoothing, up to S=22, which represents high smoothing. Not much change was observed beyond S=6.

**Figure 7:**
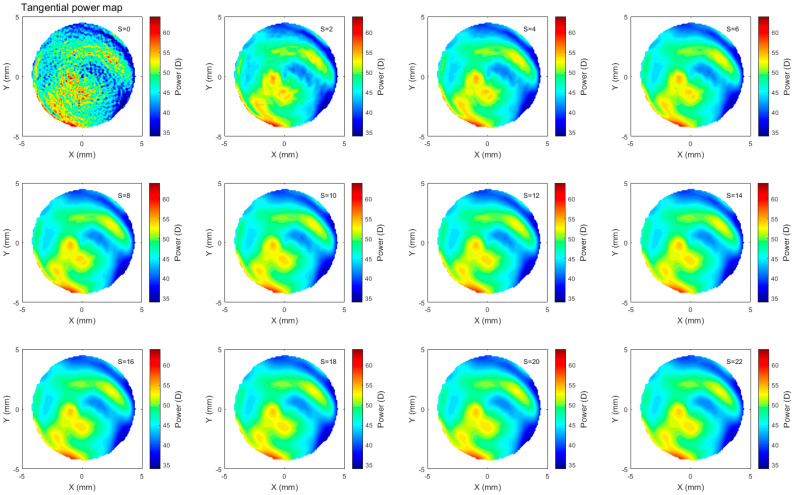
Tangential refractive power map of a rabbit’s left eye smoothed to different ranges with the scaler S changing from S=0, which represents no smoothing, to S=22, which represents high smoothing. Not much change was observed beyond S=16.

**Figure 8:**
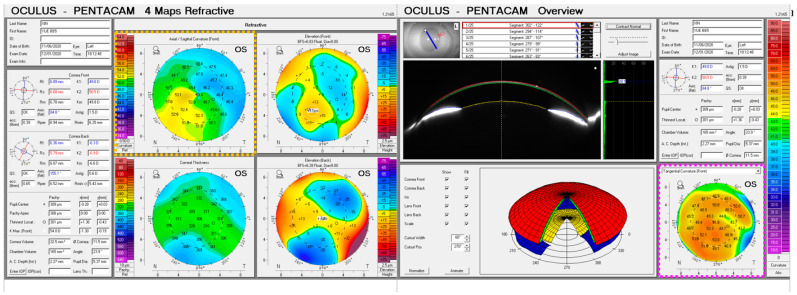
Curvature maps, as outputted by the Pentacam tomographer, for the same rabbit eye reported in Figure 6 (yellow dashed rectangle) and Figure 7 (pink dashed rectangle).

Refractive corneal power maps were produced by computing both the axial and tangential curvature from the raw Pentacam elevation data and then smoothed using varying degrees of smoothing, Figure 6 and Figure 7. When applying different degrees of smoothing to the axial curvature maps, it was noted that moving up to S=6 gave a good representation of the surface without missing any important features, as can be seen by visually comparing smoothed maps to those produced by the corneal tomographer software, Figure 8. If the same logic is applied to the tangential curvature maps, a smoothing degree of S=16 was visually identified to achieve a similar smoothness to that which is shown in the maps generated using the corneal tomographer software, Figure 6.

**Figure 9:**
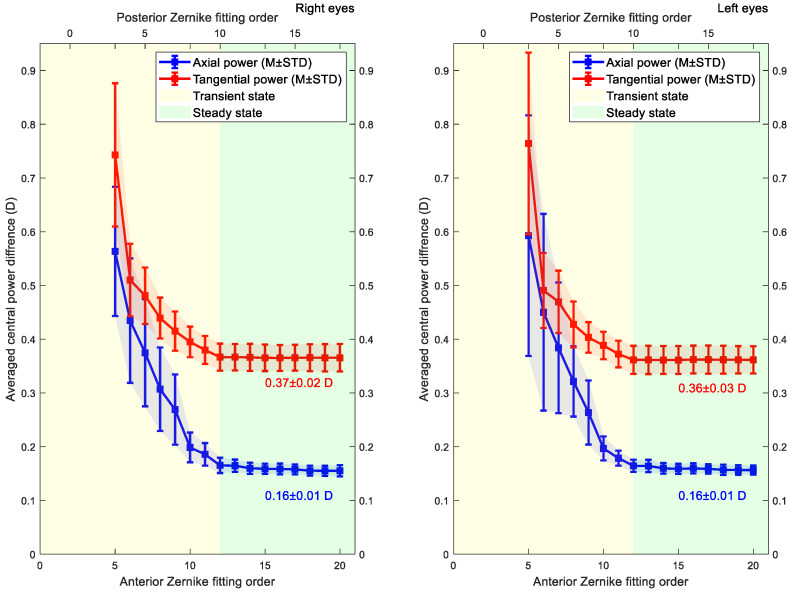
The average difference between axial and tangential power for surfaces was generated using different Zernike polynomial orders, and the power values were computed using the original elevation data. Results only consider the central optic zone of the cornea (central 3 mm diameter). The transient state orders show significant changes in power differences; however, steady state orders show a stable change in power differences.

Average central axial and tangential power differences were computed for posterior and anterior corneal elevation data fitted to Zernike polynomial with different orders, Figure 9. Average errors of the calculated power within the 3 mm central optic zone and their standard deviations were then computed. The average errors in axial and tangential refractive powers showed convergence at and after fitting order 12. Beyond this order, the errors converged to 0.16 ± 0.01 D, 0.37 ± 0.02 D in the right eyes and 0.16 ± 0.01 D, 0.36 ± 0.03 D in left eyes for the axial and tangential curvature maps, respectively.

**Figure 10:**
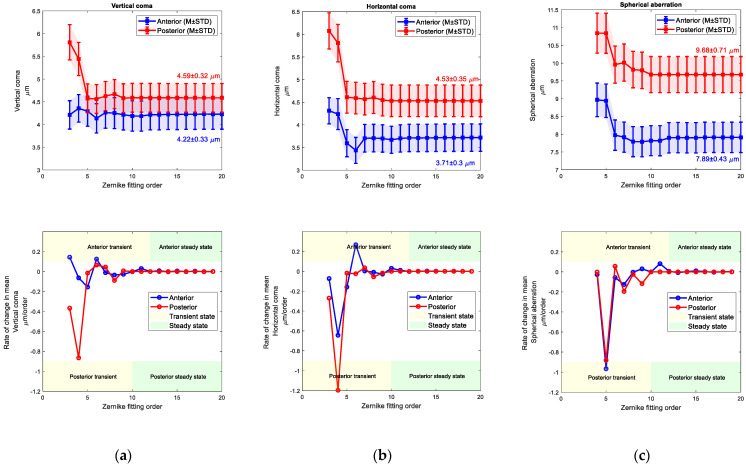
Right eyes set, (**a**) Vertical coma Zernike coefficient (**top**) and its rate of change with the fit order (**bottom**), (**b**) Horizontal coma Zernike coefficient (**top**) and its rate of change with the fit order (**bottom**), (**c**) Spherical aberration Zernike coefficient (**top**) and its rate of change with the fit order (**bottom**).

**Figure 11:**
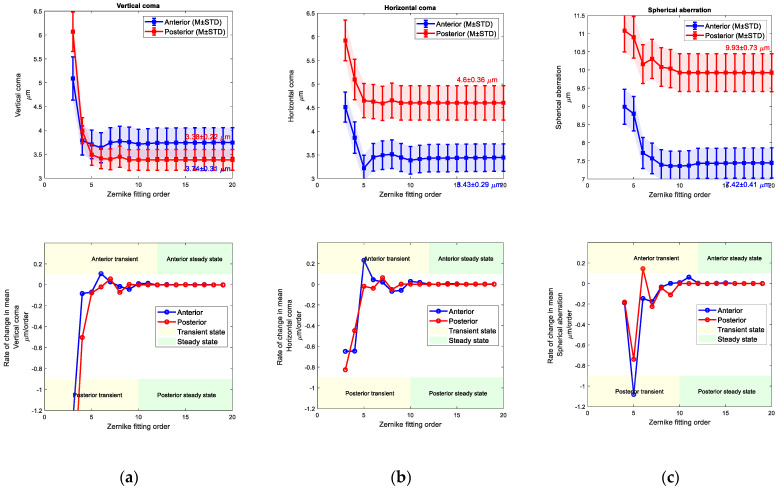
Left eyes set, (**a**) Vertical coma Zernike coefficient (**top**) and its rate of change with the fit order (**bottom**), (**b**) Horizontal coma Zernike coefficient (**top**) and its rate of change with the fit order (**bottom**), (**c**) Spherical aberration Zernike coefficient (**top**) and its rate of change with the fit order (**bottom**).

When vertical, horizontal commas and spherical aberration coefficients were tested against the Zernike order fitting, fluctuations were observed on the values in low orders (transient state), but once the order of fitting is equal or passes 12 and 10 for the anterior and posterior surfaces, the values were stalled (steady state), (see Figure 10 and Figure 11). Right eyes aberrations were settled at 4.22 ± 0.33 µm, 3.71 ± 0.3 µm, and 7.89 ± 0.43 µm while left eyes were settled at 3.74 ± 0.31 µm, 3.43 ± 0.29 µm and 7.42 ± 0.41 µm. Rates of change in fitted values of Zernike cototients were observed by the first derivative of these values, and the steady state was recognised when the rate of change was close to zero.

## 5. Discussion

Rabbit eyes are frequently used for animal-based investigations of various ocular applications because of their similarity in size to the human cornea, in addition to producing consistent and repeatable results at a low cost [22] due to the ease of manipulation [23]. They have been successfully used for assessing the implantation of intraocular lenses (IOL) [24], inlay implantation [25], corneal stromal opacity [26], laser-based vision correction [27,28], the complication of refractive surgery [29] and approving the safety of intrastromal laser ablation [30,31]. Zernike polynomials are widely used to describe the shape of the corneal surface through their terms and coefficients [5,32,33,34]. Using Zernike polynomial fitting, rabbit eyes were reported to have lower refractive errors, when compared to human eyes, but larger higher-order aberrations [35]. Geometrically, through the use of Zernike polynomials, the corneal surface can be reconstructed from the combination of terms that have a physical meaning directly connected to the characteristics of the ocular surface [36]. Optically, a light wavefront at a specific time instance is a surface that perpendicularly joins all light rays’ points generated by the same source and have the same phase. Ideally, the wavefront must be a perfect sphere centred on the source point if the light is not refracted. Zernike polynomials are widely used to describe the light wavefront over the surface of a circular pupil, hence used in showing the eye’s behaviour in spread-out light rays or the so-called eye’s aberrations. Zernike polynomials have the ability to dismantle the optical aberrations to individual components, hence, the ability to help to determine vertical and horizontal commas in addition to spherical aberration.

When measuring corneal tomography, the Pentacam uses a Scheimpflug system to take elevation measurements at several equally spaced meridians around the eye [1]. To obtain values for points in between these meridians, surface fitting or interpolation must be used. In this study, corneal tomography data measured using Pentacam were obtained from twenty-one rabbits and analysed in order to identify the optimal order of Zernike polynomials. The current study confirms the Pentacam-based measurement findings of Wei et al.’s earlier study [4] by showing that the anterior and posterior elevation data outputted by the Pentacam tomography device are optimally fitted to Zernike polynomials of order 12 and 10, respectively. This behaviour has been previously reported with both healthy and keratoconic eyes with human participants [4], and now in animal eyes as reported in this study. 

When compared to the raw elevation data, even with optimal polynomial fitting, there were residual errors of 0.54 µm and 0.49 µm for the anterior and posterior surfaces, respectively, in the right eye population and 0.52 µm and 0.49 µm, respectively, in the left eye population. For a 10 mm diameter fit of the cornea, these errors are far lower than those achieved when using a conic-fit, which was reported as 21.18 ± 11.1 µm by [37,38] performed a similar study whereby they investigated the effect of varying the order of meridional polynomial fitting on the RMS error. For a 10.7 mm diameter, their data suggested that optimal fits are obtained with fit orders of 8 or higher. These orders were able to achieve an RMS of roughly 0.08 µm, far lower than observed in this study. These results suggest that, despite the usefulness of Zernike polynomials when describing corneal shape, meridional polynomials provide the greatest accuracy, relative to the raw elevation data. 

Axial and tangential power maps computed using the raw elevation data contain noise and require smoothing for effective visualisation. Digital noise is systematically generated while processing discrete data collected during the eye scanning process. For this reason, an investigation into the impact of smoothing the resulting refractive power maps was conducted, to reduce the noise, whilst ensuring no key information is lost in the process. This analysis highlighted that the tangential curvature maps were far more sensitive to digital noise than axial refractive power maps (Figure 6 and Figure 7). Tangential curvature is calculated using the second derivative of the raw elevation data; however, axial curvature is calculated using the first derivative. This exercise demonstrated that the second derivative creates more digital noise (less signal-to-noise ratio) than the first derivative and, as a result, tangential maps need more smoothing than axial maps. The data suggests that the curvature map displayed by the tomographer software is smoothed. This is evident in the maps with minimal smoothing where the digital noise, systematically generated during the calculations, drastically reduces the practicality of using them for diagnosis.

Axial and tangential power maps were then computed using corneal surface data obtained from Zernike polynomials of varying order. The results show that reconstructing the corneal surface through the use of Zernike polynomials induces errors in the calculation of corneal refractive power. This is due to the loss of accuracy during the fitting process itself and the existence of the systematic digital noise associated with calculating both axial and tangential curvatures. Therefore, getting the same refractive corneal power from a Zernike reconstructed surface cannot be achieved. Users need to acknowledge that reconstructing refractive power maps through Zernike polynomials will incur a loss in a portion of these powers as a residual error. However, they can minimise these residual powers by using Zernike polynomials with orders of at least 12 and 10 when fitting the anterior and posterior surfaces, respectively. Even with these optimal Zernike orders, there will still be errors of around 0.16 D and 0.36 D when computing the axial and tangential power, respectively, although these errors are not clinically significant. 

## 6. Conclusions

The current study evaluated Zernike fitting in rabbit corneas using a reverse engineering approach in attempts to utilise deductive reasoning to understand how Pentacam device software performs. The result confirms that the optimal Zernike orders for fitting to Pentacam-measured tomography data are 12 and 10 for the anterior and posterior surfaces, respectively. Axial and tangential power maps were computed using raw elevation and Zernike polynomial fitted data. In doing so, the necessity of smoothing for practical purposes was demonstrated. It was also demonstrated that reconstructing corneal surfaces using Zernike polynomials induces a residual error in the calculation of axial and tangential refractive power. The aforementioned optimal Zernike polynomial orders were able to minimise this error, although residual errors of 0.16 and 0.36 D were still present for the axial and tangential curvature maps, respectively. Each of these results is important when considering the precision of the tomographic or power map data, something that is influential in several clinical applications, such as keratoconus progression and ectasia screening [39,40]. 

Ultimately, the Pentacam utilises the Scheimpflug principle by taking either 25 (standard settings) or 50 (high resolution (HR) settings) scans in two seconds as its camera rotates around its axis, dealing with potential eye movement and discrete images requires full reconstruction of the surface raw elevation. As the calculation of curvatures from reconstructed elevation has severe resolution requirements, polynomial-based smoothing appeared to be a proper option. The current study findings support the hypothesis that Pentacam eye anterior and posterior surfaces are fitted to order 12 and 10 Zernike polynomials, respectively within the DSP implemented in the Pentacam software, as rabbit eyes showed an identical fit performant that is similar to the human eyes [4]. This identicality was observed regardless of the systematic misalignment errors associated with capturing rabbit eyes’ tomography. Finally, to get stable values of high-order aberrations that are not affected by Zernike polynomials, such as commas for ICRS adjustments [9] or spherical aberration for pre-cataract operations [10], the current study recommends using order 12 and 10 Zernike polynomials specifically to fit corneal anterior and posterior surfaces, respectively, as long as the Pentacam is being used as a tomographer in the measurement process. This conclusion should not be applied interchangeably with other eye tomography or topography instruments due to variations in their measurement methods and associated DSP procedures.

## Data Availability

The data that support the findings of this study are available on request from the corresponding author.

## References

[1] Ceylan O.M., Turk A., Erdurman C., Mumcuoglu T., Erdem U., Gokce G., Dagli S. (2011). Comparison of Oculus Pentacam and Stratus Optical Coherence Tomography for Measurement of Central Corneal Thickness. Cornea.

[2] Mohammadpour M., Heidari Z., Mohammadpour M. (2021). Pentacam. Diagnostics in Ocular Imaging: Cornea, Retina, Glaucoma and Orbit.

[3] Fan R., Chan T.C., Prakash G., Jhanji V. (2018). Applications of corneal topography and tomography: A review. Clin. Exp. Ophthalmol..

[4] Wei Y., Lopes B.T., Eliasy A., Wu R., Fathy A., Elsheikh A., Abass A. (2021). Performance of Zernike polynomials in reconstructing raw-elevation data captured by Pentacam HR, Medmont E300 and Eye Surface Profiler. Heliyon.

[5] Zernike V.F. (1934). Beugungstheorie des schneidenver-fahrens und seiner verbesserten form, der phasenkontrastmethode. Physica.

[6] Harvey H.B., Sotardi S.T. (2018). The Pareto Principle. J. Am. Coll. Radiol..

[7] Fathy A., Lopes B.T., Ambrósio R., Wu R., Abass A. (2021). The Efficiency of Using Mirror Imaged Topography in Fellow Eyes Analyses of Pentacam HR Data. Symmetry.

[8] Consejo A., Fathy A., Lopes B.T., Ambrósio R., Abass A. (2021). Effect of Corneal Tilt on the Determination of Asphericity. Sensors.

[9] Kang M.-J., Byun Y.-S., Yoo Y.-S., Whang W.-J., Joo C.-K. (2019). Long-term outcome of intrastromal corneal ring segments in keratoconus: Five-year follow up. Sci. Rep..

[10] de Sanctis U., Vinai L., Bartoli E., Donna P., Grignolo F. (2014). Total spherical aberration of the cornea in patients with cataract. Optom. Vis. Sci..

[11] Olsen T. (1986). On the calculation of power from curvature of the cornea. Br. J. Ophthalmol..

[12] Ho J.-D., Tsai C.-Y., Tsai R.J.-F., Kuo L.-L., Tsai I.L., Liou S.-W. (2008). Validity of the keratometric index: Evaluation by the Pentacam rotating Scheimpflug camera. J. Cataract Refract. Surg..

[13] Smit G., Atchison D.A. (1970). The Eye and Visual Optical Instruments.

[14] Vojnikovi B.o., Tamajo E. (2013). Gullstrand’s Optical Schematic System of the Eye Modified by Vojnikovi & Tamajo. Coll. Antropol..

[15] KLEIN S.A. (1997). Axial Curvature and the Skew Ray Error in Corneal Topography. Optom. Vis. Sci..

[16] Clark V.L., Kruse J.A. (1990). Clinical Methods: The History, Physical. JAMA.

[17] Walker H.K., Hall W.D., Hurst J.W. (1990). Clinical Methods: The History, Physical, and Laboratory Examinations.

[18] Garcia D. (2010). Robust smoothing of gridded data in one and higher dimensions with missing values. Comput. Stat. Data Anal..

[19] Abass A., Clamp J., Bao F., Ambrosio R., Elsheikh A. (2018). Non-Orthogonal Corneal Astigmatism among Normal and Keratoconic Brazilian and Chinese populations. Curr. Eye Res..

[20] Doll T., Moore J., Shihab A.H., Lopes B.T., Eliasy A., Maklad O., Wu R., White L., Jones S., Elsheikh A. (2020). Which feature influences on-eye power change of soft toric contact lenses: Design or corneal shape?. PLoS ONE.

[21] Everitt B.S., Skrondal A. (2010). The Cambridge Dictionary of Statistics.

[22] Marcos S. (2006). Aberrometry: Basic science and clinical applications. Bull. Soc. Belg. Ophtalmol..

[23] Yüksel H., Türkcü F.M., Ari Ş., Çinar Y., Cingü A.K., Şahin M., Şahin A., Özkurt Z., Çaça İ. (2015). Anterior segment parameters of rabbits with rotating Scheimpflug camera. Vet. Ophthalmol..

[24] Robinson M.L. (2009). Animal Models in Eye Research. Hum. Genom..

[25] Kim E., Ehrmann K., Choo J., Franz S., Moilanen J. (2013). The Effect of Inlay Implantation on Corneal Thickness and Radius of Curvature in Rabbit Eyes. Cornea.

[26] Joshi V.P., Vaishnavi K.S., Ojha S.K., Singh V., Basu S. (2020). A reliable animal model of corneal stromal opacity: Development and validation using in vivo imaging. Ocul. Surf..

[27] Peyman G.A., Badaro R.M., Khoobehi B. (1989). Corneal ablation in rabbits using an infrared (2.9-microns) erbium: YAG laser. Ophthalmology.

[28] Pallikaris I.G., Papatzanaki M.E., Stathi E.Z., Frenschock O., Georgiadis A. (1990). Laser in situ keratomileusis. Lasers Surg. Med..

[29] Smith R.J., Maloney R.K. (1998). Diffuse lamellar keratitis: A new syndrome in lamellar refractive surgery11The authors have no proprietary interest related to this article. Ophthalmology.

[30] Zhang Z.-Y., Chu R.-Y., Zhou X.-T., Dai J.-H., Sun X.-H., Hoffman M.R., Zhang X.-R. (2009). Morphologic and Histopathologic Changes in the Rabbit Cornea Produced by Femtosecond Laser–Assisted Multilayer Intrastromal Ablation. Investig. Ophthalmol. Vis. Sci..

[31] Nishi O., Hara T., Hara T., Sakka Y., Hayashi F., Nakamae K., Yamada Y. (1992). Refilling the lens with a inflatable endocapsular balloon: Surgical procedure in animal eyes. Graefes Arch. Clin. Exp. Ophthalmol..

[32] Khorin P., Ilyasova N., Paringer R.A. (2018). Informative feature selection based on the Zernike polynomial coefficients for various pathologies of the human eye cornea. Comput. Opt..

[33] McAlinden C., Schwiegerling J., Khadka J., Pesudovs K. (2020). Corneal aberrations measured with a high-resolution Scheimpflug tomographer: Repeatability and reproducibility. J. Cataract Refract. Surg..

[34] Schröder S., Mäurer S., Eppig T., Seitz B., Rubly K., Langenbucher A. (2018). Comparison of Corneal Tomography: Repeatability, Precision, Misalignment, Mean Elevation, and Mean Pachymetry. Curr. Eye Res..

[35] Chen L., Huang L.C., Gray B., Chernyak D.A. (2014). Comparison of wavefront aberrations in rabbit and human eyes. Clin. Exp. Optom..

[36] Bouazizi H., Brunette I., Meunier J. Are There Categories of Corneal Shapes?. Proceedings of the 2018 40th Annual International Conference of the IEEE Engineering in Medicine and Biology Society (EMBC).

[37] Read S.A., Collins M.J., Carney L.G., Franklin R.J. (2006). The topography of the central and peripheral cornea. Investig. Ophthalmol. Vis. Sci..

[38] Franklin R.J., Morelande M.R., Iskander D.R., Collins M.J., Davis B.A. (2006). Combining Central and Peripheral Videokeratoscope Maps to Investigate Total Corneal Topography. Eye Contact Lens Sci. Clin. Pract..

[39] Meyer J.J., Gokul A., Vellara H.R., Prime Z., McGhee C.N.J. (2017). Repeatability and Agreement of Orbscan II, Pentacam HR, and Galilei Tomography Systems in Corneas with Keratoconus. Am. J. Ophthalmol..

[40] Kosekahya P., Koc M., Caglayan M., Kiziltoprak H., Atilgan C.U., Yilmazbas P. (2018). Repeatability and reliability of ectasia display and topometric indices with the Scheimpflug system in normal and keratoconic eyes. J. Cataract Refract. Surg..

